# Aircraft Detection in High-Resolution SAR Images Based on a Gradient Textural Saliency Map

**DOI:** 10.3390/s150923071

**Published:** 2015-09-11

**Authors:** Yihua Tan, Qingyun Li, Yansheng Li, Jinwen Tian

**Affiliations:** 1 School of Automation, Huazhong University of Science and Technology, Luoyu Road 1037, Hongshan District, Wuhan 430074, China; E-Mails: yhtan@hust.edu.cn (Y.T.); caobaoyun_good@126.com (Q.L.); jwtian@hust.edu.cn (J.T.); 2 School of Remote Sensing and Information Engineering, Wuhan University, Luoyu Road 129, Wuchang District, Wuhan 430079, China

**Keywords:** synthetic aperture radar (SAR), aircraft detection, directional local gradient distribution, gradient textural saliency map

## Abstract

This paper proposes a new automatic and adaptive aircraft target detection algorithm in high-resolution synthetic aperture radar (SAR) images of airport. The proposed method is based on gradient textural saliency map under the contextual cues of apron area. Firstly, the candidate regions with the possible existence of airport are detected from the apron area. Secondly, directional local gradient distribution detector is used to obtain a gradient textural saliency map in the favor of the candidate regions. In addition, the final targets will be detected by segmenting the saliency map using CFAR-type algorithm. The real high-resolution airborne SAR image data is used to verify the proposed algorithm. The results demonstrate that this algorithm can detect aircraft targets quickly and accurately, and decrease the false alarm rate.

## 1. Introduction

The ability to provide high resolution images of synthetic aperture radar (SAR) in any weather and at any time is extremely important for disaster monitoring, modern military reconnaissance and combat tasks. However, the special characteristics of SAR imaging process, speckle noise, and the high-variability of man-made targets due to the change of azimuth, imaging parameters, shade, background environment and so on, lead to a huge challenge in the SAR image target detection field [1,2]. There is no omnipotent detector that can perform well under all different conditions, which was pointed out by Cui [3]. Thus, it is very crucial to elaborately devise an algorithm to detect targets accurately and robustly from SAR images for a specific application scenario even though a unified framework is strongly desired. It is still an open issue to propose the target detection algorithm by considering the SAR image features and application contextual cues.

Target detection in a SAR image involves namely extracting the targets of interest from a scene with a background which usually is contaminated by heavy noise. This problem has been studied by many researchers until now. Based on the analysis of [4], the methods of target detection from SAR images can be broadly classified into three types: single-feature based, multi-feature based, and expert-system-oriented.

For the single-feature based category in which only brightness feature is utilized, the Constant False Alarm Rate (CFAR)-based methods are widely exploited in the literature because the CFAR methods have the properties of simple computing, constant false alarm, adaptive threshold, and fast speed of extracting targets from the complex background [5,6,7]. CFAR methods have been applied to many SAR Automatic Target Recognition (ATR) systems [8,9]. The variants of CFAR algorithm include the cell averaging CFAR (CA-CFAR) [10], greatest of CFAR (GO-CFAR), smallest of CFAR (SO-CFAR) [11], order statistic CFAR (OS-CFAR) [12], Variability Index CFAR (VI-CFAR) [13]. In addition, there are also some new CFAR detectors, such as stepwise accumulation cell averaging (SCCA-CFAR) detector [14], two level CFAR detector (TL-CFAR) [15] and so forth. VI-CFAR adaptively chooses CA-CFAR, SO-CFAR or GO-CFAR to cope with different background distribution. Therefore, it can achieve better performance than CA-CFAR, SO-CFAR or GO-CFAR. SCCA CFAR has the most complex computation. TL-CFAR detector attempts to apply integral image to reduce the computational complexity. Even though great efforts have been made to keep the targets intact, the detected targets are usually separated into points or blocks due to the speckle noise and special SAR imaging process. To mitigate this problem, K-means clustering was adopted to group the separated regions into an intact target [16]. However, this approach [16] is sensitive to the target size and clustering threshold even though it can preserve more structural features for the following recognition step. In addition to CFAR-based methods, there are several other non-CFAR methods. The detector proposed by Ouchi *et al.* [17] extracts the targets from the coherence image which is the cross correlation of two multi-look SAR images. This method can detect objects buried in strong speckle noises. Howard *et al.* [18] presented a detector by exploiting genetic programming. These methods were devised for detecting small target with several or tens of pixels so that they are not suitable for airplane detection in high-resolution SAR images in which the target may contains several hundreds of pixels.

For the multi-feature based category, the algorithms try to extract additional features, such as variance features, fractal features, and wavelet features [19,20]. As the third category, expert-system-oriented algorithms incorporate the prior information or intelligent preprocessing into the detection framework. Xie *et al.* proposed a detection algorithm based on visual saliency [21]. However, this algorithm is sensitive to the window size, and it can only obtain better results than CFAR algorithm on the condition that the size of the target is fixed. Gao *et al.* proposed a vehicle target detection algorithm with SAR images based on contextual knowledge [22]. It combines prior information (*i.e.*, terrain types, distances to boundary and target aggregation degree) and the result of Markov Random Field (MRF) based segmentation as the initial guess for improved CA-CFAR. These algorithms perform well in particular situation when the targets are small and not separated into many blobs.

In our application case of aircraft detection from high-resolution SAR image, some special properties should be taken into account. First, an airplane target consists of bright pixels caused by strong scattering points and dark pixels generated by weak scattering points in some places of the target. This property indicates that we can extract the candidate regions as the potential target location according to brightness. Then we may segment the intact target by grouping the bright and dark pixels of a target in the local candidate region. As mentioned above, it is hard to group the broken parts of aircraft to an intact one if we only segment the pixels separately in SAR image. However, these dark and bright pixels form a special feature of salient structure texture, which can be exploited to perceptually connect the target blobs. Second, the contextual information that the aircraft targets always park on the apron area can be utilized so that we only need to detect the aircraft targets in candidate apron region. Because segmentation of apron region in high resolution SAR image is comparatively easy job, the utilization of contextual knowledge is very critical to reduce false alarm rate and computational complexity.

Visual saliency is the noticeable perceptual property which makes the targets in the visual field pop out and catches our attention. In the computer vision field, a lot of works were conducted on detecting targets by analyzing the saliency map in image. As the core part of such work, the measurement of saliency is very crucial, which tells us which part of an image is salient. For remote sensing application, Hu *et al.* proposed local edge distributions (LED) for detection of salient structure textures [23]. Motivated by this, we propose Gradient Textural Saliency (GTS) map to represent the likelihood of a pixel being target by using directional local gradient distribution. Then we can segment the intact aircraft target from the saliency map. To fully make use of the above second property, all the saliency maps are only generated on the candidate apron regions to improve the detection accuracy and reduce computational complexity as the strategy taken by Gao *et al.* [22] to exploit the prior knowledge.

Therefore, the proposed aircraft detection algorithm consists of four steps: (1) extracts the apron areas using segmentation; (2) generates gradient image in apron areas; (3) produce gradient textural saliency map with directional local gradient distribution algorithm in the candidate regions; (4) use a more precise method—TL-CFAR to detect aircraft target from the saliency map. As a whole, the novelties of the algorithm include the following aspects:

- Contextual cue is integrated into the algorithm. Apron regions where the airplanes park are generally dark homogenous so that they are easily extracted from the whole image of airport.
- The concept of gradient textural saliency map is introduced. As analyzed by Hu *et al.* [23], each target has edge saliency in the optical image. However, edges of a target in SAR image are not very clear because the pixels are the sampling of backscattering blobs whose strengths usually vary severely along a target. Even so, the target in SAR image is still salient in gradient domain by measuring the gradient textural saliency.
- Directional local gradient distribution is proposed to measure the textural saliency of a target, which we call as GTS. The saliency of each pixel is represented by an index which is computed by local gradient point density in a local window. This method is able to connect the dark and bright pixels of a target by imposing the interactive constraints between the neighboring pixels.

The remainder of this paper is organized as follows. Section 2 first introduces gradient textural saliency map. On the basis of gradient textural saliency map, Section 3 presents the aircraft detection algorithm. Section 4 gives a comprehensive analysis of the proposed aircraft target detection approach. Section 5 gives a conclusion for this paper.

## 2. Gradient Textural Saliency Map

Since a SAR image is the sampling of the backscattering coefficient, CFAR-based algorithms are naturally adopted to determine if a pixel belongs to a target in terms of statistical computation. These methods output isolated discriminative results without considering the relationship of neighboring pixels in a target. Thus, the targets extracted by CFAR-based methods must be processed further by clustering or morphological steps in order to prepare intact objects for recognition. However it is not easy to get this job done because of the loss of much original information. In the computer vision field, salient object detection is becoming one of the mainstream generic object detection methods [24,25]. The key idea is to build the saliency map using local contrast. Hu *et al.* proposed local edge distributions for detection of salient structure textures for optical remote sensing image [23]. The algorithm is not suitable for our application because it is difficult to extract accurate and clear edges from SAR image. Xie *et al*. also presented a detection algorithm in SAR image motivated by the idea of visual saliency [19]. However, it is not genuine salient detection algorithm from the perspective of visual saliency since the saliency of a pixel is measured following the idea of CFAR.

Before we present the details of our algorithm, we first explain the concept of gradient textural saliency map. As we know, our attention to an object in the visual field is just because the object is significantly distinct from the surroundings, which is called as visual saliency defined in contrast based on various types of image features. For optical images, there are a lot of definitions of contrast measures to create different visual saliency maps [26]. Few works were conducted that were concerns about the definition of visual saliency by considering the special characteristics of SAR image.

The targets in SAR images consist of strong scattering spots, which may emerge as several pixels. Normally, an airplane target is not as intact as the appearance shown in the optical image. The bright pixels can be perceived as part of an airplane just because the fragments of the target have visual saliency. However, taking the texture of SAR image into account, we can segment the enclosed target region even though there are holes and clearance among the target fragments. Specifically, the texture feature in gradient domain of a target in SAR image makes target region salient from the background. Following the framework of salient object detection [24,25], the first step is to build the saliency map of the image. Therefore, we name the saliency map in gradient domain as Gradient Textural Saliency (GTS) Map.

**Figure 1:**
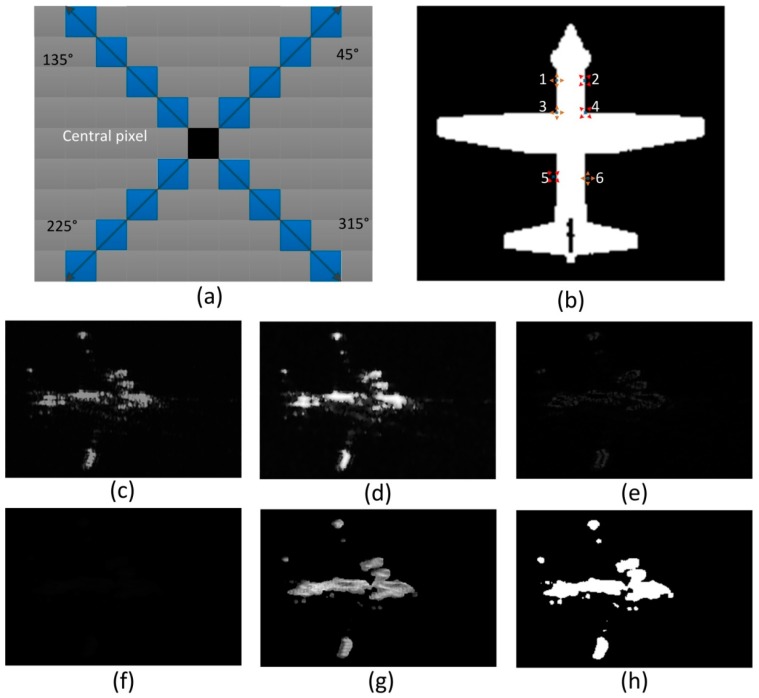
Sketch map of the salient target detection from gradient textural saliency map. (**a**) the sketch map of 4-direction window; (**b**) the sketch map of 4-direction window on target; (**c**) the original image; (**d**) the pre-processed image of (**c**); (**e**) the gradient image of (**d**); (**f**) the gradient textual saliency map of (**e**) (it shows like black image because the index values of pixels are very small); (**g**) the normalized saliency map of (**f**) according to Equation (12) in Section 3.2; (**h**) the segmented binary image of (**g**).

Even though SAR image is not genuine optical image, the neighboring pixels still have close connections such as similar brightness or gradient. To utilize this locality, we define textural saliency of a pixel as an index computed in the gradient domain in a local window. Specifically, the textural saliency index of a pixel is defined on the basis of the gradient distribution in the local window. In order to make the distribution representative and avoid the impact of noise, we choose a local 4-direction window with interval of 90° between the two neighboring directions, shown as Figure 1a. We name the gradient distribution on the four directions as Directional Local Gradient Distribution. The gradient saliency indices of all the pixels build a gradient textural saliency map of target candidate region. Then the segmentation method is applied to extract the salient airplane target. Figure 1 shows the basic concept and computation procedure of the proposed gradient textural saliency map. In addition, Figure 1c–g explains the process of the generation of the gradient textural saliency map.

From Figure 1, we can visually understand the basic concept of the gradient textural saliency map. Figure 1a shows the local 4-direction window which includes four directions of 45°, 135°, 225°, 315°. Figure 1b shows the sketch map of two kinds of local 4-direction window on an airplane. Window 1, 3, 6 have 4-direction of 0°, 90°, 180°, 270°, and window 2, 4, 5 have 4-direction window of 45°, 135°, 225°, 315°. Figure 1c shows a real SAR image contains one aircraft target. Figure 1d shows the image after divided linear stretching and speckle suppression on Figure 1c. Figure 1e shows the original gradient image of Figure 1d. Figure 1f shows the gradient textual saliency map of Figure 1e. Figure 1g shows the gradient textual saliency map of Figure 1f after normalization. In addition, Figure 1h shows the binary image after segmentation on Figure 1f based on the TL-CFAR method. From Figure 1, we can see that we can extract an intact aircraft with its most parts are interconnected by using gradient textural saliency map.

### 2.1. Procedure of Saliency Map Generation

**Figure 2:**
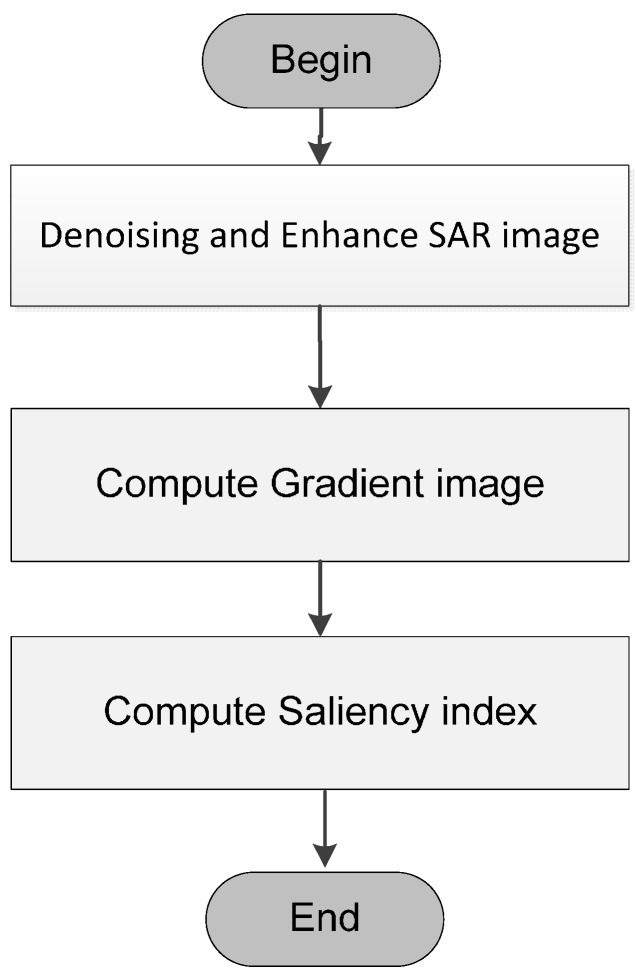
Salient target detection and textural saliency map generation based on directional local gradient distribution.

As shown as Figure 2, the generated map is used as the input of salient target detection module. Firstly, we implement the preprocessing modules, including suppressing speckle noise using Frost filter [27] and enhancing the brightness and contrast of image with adaptive piecewise linear gray level stretch algorithm [28]. Secondly, we extract the original gradient image from the enhanced SAR image. Thirdly, we compute the gradient saliency indices of all the pixels by traversing the gradient image using local 4-direction window proposed in this paper. In addition, we will generate a gradient textural saliency map which is segmented to extract the salient targets.

### 2.2. Generation of Gradient Image

With the preprocessed image by using divided linear stretch and Frost filter to suppress the speckle noise, we extract the horizontal and vertical gradient of the image by differential mean value based on the two neighbors of a pixel firstly. Then, we compute the magnitude of all the pixels by the horizontal and vertical gradient. For a SAR image with size of *m* × *n*, the computation formulas are shown in the following [28]:

(1) $$G\left(i,j\right)=\sqrt{G\left(h\right){\left(i,j\right)}^{2}+G\left(v\right){\left(i,j\right)}^{2}}$$

where G(h)(i,j)=[I(i+1,j)−I(i−1,j)]/2, G(v)(i,j)=[I(i,j+1)−I(i,j−1)]/2, I(i,j) is the brightness of a pixel (i,j) in the preprocessed SAR image. G(h)(i,j) is the horizontal gradient of pixel (i,j), G(v)(i,j) is the vertical gradient of pixel (i,j).

Finally, we can produce the original gradient image here. Figure 1e shows the original gradient image generated from Figure 1d.

### 2.3. Gradient Textural Saliency (GTS) Map Generation Based on Directional Local Gradient Distribution

A local 4-direction window will be used to traverse all gradient points in the original gradient image generated in last step. As the local window is separated into four directions with the processed pixel as the center, the gradient textural saliency index of the pixel is computed based on the density of local gradient and the distribution evenness of the gradients in the local 4-direction window. Saliency index of the center pixel $\left({\text{i}}_{0},{\text{j}}_{0}\right)$ is defined as following:

(2) $$G_{s}=G_{de}\cdot G_{ev}$$

where “·” denotes multiplication and,

(3) $$G_{de}=\left[SUM_{g}/N+G\left(i_{0},j_{0}\right)\right]/2$$

(4) $$G_{ev}=\frac{n_{\text{θ}}\cdot \left(G_{max}+G_{min}\right)/2}{SUM_{g}}$$

with

(5) $$N=n_{\text{θ}}\cdot L$$

(6) $${SUM}_{g}=\overset{n_{\text{θ}}}{\underset{\text{α}=1}{\sum }}\overset{L}{\underset{\text{β}=1}{\sum }}G\left(i^{\prime },j^{\prime }\right)$$

(7) $$i^{\prime }=i_{0}-\text{ β}\cdot sin\text{θ}$$

(8) $$j^{\prime }=j_{0}+\text{ β}\cdot cos\text{θ}$$

where $G_{s}$ represents the gradient textural saliency index. $G_{de}$ represents the local gradient density computed by the Equation (3) and $G_{ev}$ represents the local gradient distribution evenness computed by the Equation (4) which is used to measure how evenly the gradients are distributed in four directions, $n_{\text{θ}}$ is the direction number. ${SUM}_{g}$ indicates the sum of gradients in the local 4-direction window. *N* represents the total number of pixels in the local directional window. $G\left(i_{0},j_{0}\right)$ is the gradient of the center pixel of the window. $G_{max}$ and $G_{min}$ represent the maximum gradient value and the minimum gradient value in the window respectively. *L* is the length of each direction, here we also call it radius of local 4-direction window. $\left(i^{\prime },j^{\prime }\right)$ is the coordinate of a pixel in the four direction. θ is the direction angle. If we preset the beginning direction angle ${\text{θ}}_{0}$, and the number of directions, we can use Equation (9) to compute the adjacent angle interval and use Equation (10) to compute the next direction angle θ.

(9) $$\text{γ}=360/n_{\text{θ}}$$

(10) $$\text{θ}={\text{θ}}_{0}+\left(\text{α}-1\right)\cdot \text{γ}$$

All the pixels’ saliency indices construct the textural saliency map which conveys the significance of each pixel. Meanwhile, the smoothness of neighboring pixels is also embedded in this map. Then we can use segmentation algorithm on this map to detect salient objects.

## 3. Aircraft Detection Algorithm

This paper concentrates on the detection of airplane from SAR image, which means that the airplane must park on the airport. Therefore, we can only conduct the salient target detection in the candidate region of apron. With this cue, we can utilize the significant visual discriminative feature to improve the detection accuracy and reduce the false alarm. On the other hand, the precise extraction of airplane from the saliency map is also the key point of the algorithm.

### 3.1. Extraction of Candidate Regions

**Figure 3:**
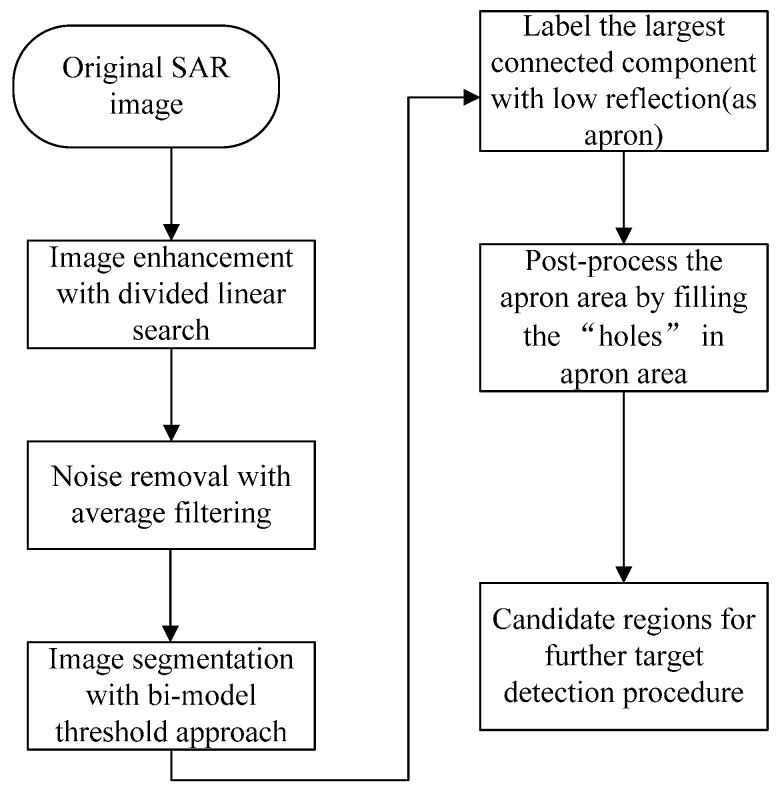
Detection procedure of apron and candidate regions map.

If we look at the airport in SAR image, we will find that apron is the most salient big area where the airplanes locate. Generally, apron is the largest continuous, homogeneous and low-reflection region in SAR images. Thus, apron is darker than the other area in the SAR airport images. Furthermore, airplane forms a hole in the extracted apron area if we use gray-level based segmentation. This implies that we can further refine the target only in this bounding rectangle of the hole. Thus, the computational burden decreases and the precision of detected target increases. We call the bounding rectangle as candidate region. In addition, we only calculate the saliency index in such regions while the saliencies of other pixels in apron area are evaluated as 0. The apron area is extracted using region segmentation and component analysis based algorithm which was described in [16]. The entire details of apron and candidate regions detection procedure in high-resolution SAR images is illustrated in Figure 3.

In this paper, we enhance the brightness and contrast of the image to highlight the image details by the adaptive piecewise linear gray level stretch algorithm [28] and suppress the speckle noise by using average filter with a window size of 5 × 5. The bimodal method [29] is adopted to segment the image. After all the steps, we can get the relatively accurate apron area and the candidate regions.

#### 3.1.1. Binary Candidate Regions Map

After the step of labelling the largest low reflection and connected component, we can get the binary candidate regions map, where the regions are shown as holes on apron area with value of 0. However, if we want to locate the candidate regions’ pixels, we firstly need to locate the pixels on the apron area accordingly.

#### 3.1.2. Relatively Accurate Apron Map

**Figure 4:**
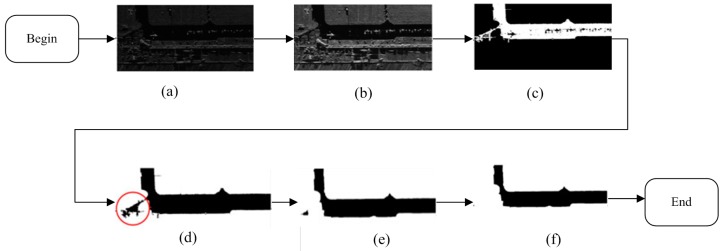
The intermediate results of the apron and holes-on-apron detection. (**a**) is the original real SAR image for example; (**b**) is the preprocessed image after enhancing image and suppressing speckles in (**a**); (**c**) is the sketch map of candidate region map after segmenting image by bimodal method, and labeling the largest connected component on (**b**), the black regions on white apron area is the candidate regions; (**d**) is the original apron area image after filling holes on apron area (candidate regions) on (**c**); (**e**) is the dilated image after applying binary-morphologically dilating on (**d**); (**f**) is the final more precise apron area after doing fill holes on (**e**) to eliminate small regions which are not on apron area.

After the step of filling “holes” on apron area, we can get an original apron map. However, it may still have some branches which will influence the detection. These branches, such as the red circle labeling area in Figure 4d, may contain some small holes which will be regarded as candidate regions in aircraft detection procedure. If we do not eliminate them, they may become false alarm in the final detection result. Therefore, here we use binary morphology dilation to eliminate the scattered and small branch structures. After binary morphology dilation, there will still be some small areas, then we execute the step of filling holes on the apron area and invert image to get a relatively accurate apron map.

Figure 4 shows the procedure results of the apron and candidate regions detection. In the experiment, the compressibility factor α in the image enhancing step is 1%; the window size of the average filter is 5 × 5. In the step of binary morphology expansion, the width and height of the window is respectively 50 and 50, and the used element shape is rectangle rather than cross by consideration of both effectiveness and reduction of computation complexity. The intermediate results and final apron result are shown in Figure 4.

From Figure 4, we can see that the apron marked image Figure 4f which is detected by using our improved method is more precise than Figure 4d. If we use Figure 4d and the candidate region map Figure 4c in the aircraft target detection step, fake targets will be detected in the area which is marked by a red circle in Figure 4d. Therefore, our apron and candidate region map detection method can get a better result, and this result can help to reduce the false alarm in the next target detection. In addition, the apron image and candidate region map will be applied to the steps of gradient image generation and gradient textural saliency map computation to largely reduce the computation requirements.

### 3.2. Salient Objects Detection Based on Gradient Textural Saliency Map

In the part, when we generate the gradient textural saliency map, we do some special handling for SAR aircraft situation in order to exploit the contextual information. Firstly, when we generate the original gradient image, we conduct the following equation:

(11) $$G\left(i,j\right)=\{\begin{array}{c} \sqrt{G\left(h\right){\left(i,j\right)}^{2}+G\left(v\right){\left(i,j\right)}^{2}}\;,\;if\;pixel\left(i,j\right)in\;apron\\ 0\;,\;else \end{array}$$

If (i ,j) pixel is in the apron area, we will compute its gradient value as Equation (11), else if the point is not on the apron area, we take its gradient value as 0 for reducing the amount of calculation.

Secondly, we compute the gradient textural index by centering the local 4-direction window on one pixel. If the pixel comes from the detected hole in the apron, then calculate its index according to Equation (2); else, set its index as zero.

However, we cannot see the targets in the original gradient textural saliency map because the pixel values of the original saliency map are very small. Here we use mean-variance gray scale normalization method to normalize the gray scale to enhance the saliency map. The normalization formula is shown as following:

(12) $${G_{s}}^{\prime }\left(i,j\right)=\left(G_{s}\left(i,j\right)-G_{s_{mean}}\right)/G_{s_{var}}$$

where

$$G_{s_{mean}}=\frac{1}{w\times h}\overset{w,h}{\underset{i=0,j=0}{\sum }}G_{s}\left(i,j\right)$$

$$G_{s_{var}}=\;\frac{1}{w\cdot h-1}\sqrt{\overset{w,h}{\underset{i=0,j=0}{\sum }}{\left(G_{s}\left(i,j\right)-G_{s_{mean}}\right)}^{2}}$$

$\;G_{s}\left(i,j\right)$ is the index value of the pixel that locates at (i,j) in the saliency image. w,h are the width and height of the image respectively. $G_{s_{mean}}$, $G_{s_{var}}$ are the mean and variance of the image respectively. After normalization step according to Equation (12), we can get the final saliency map of the targets.

This method can preserve precise and rich structural and textual feature of the aircraft target, and it can also keep the whole object intact to the greatest extent. These properties make this method have a great advantage in SAR aircraft detection. An example of the generation of the normalized saliency map is shown in Figure 1g. We can see that, even though the gradient image contain lots of false alarms, the gradient textural saliency map can reduce much of them by its special processing procedure. In addition, meanwhile, gradient textural saliency map can help to ensure the integrity of the target.

In this paper, we use TL-CFAR to segment the gradient textural saliency map to get a more precise result, because TL-CFAR detection algorithm considers both the background distribution and the local structural of the targets. The segmentation example is shown as Figure 1h.

### 3.3. Overview of Algorithm

**Figure 5:**
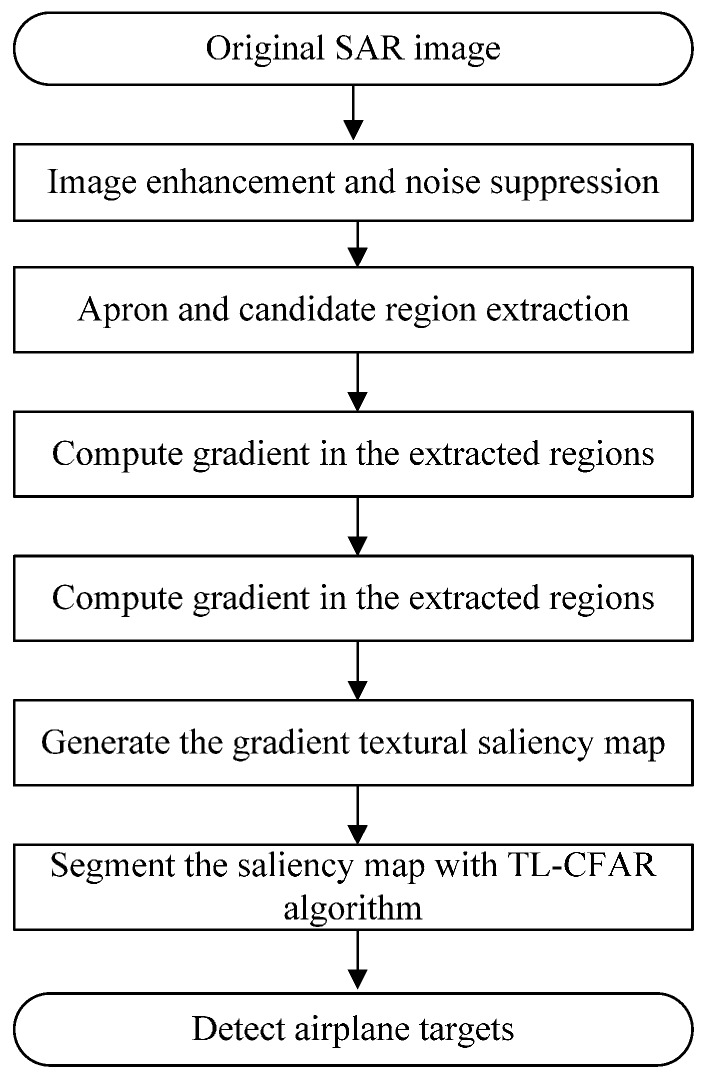
Aircraft detection algorithm proposed in this paper.

Until now, CFAR detectors are commonly used methods for detecting the targets in SAR image. Thus, we adopt the CFAR-type detector to extract the airplane targets from the produced textural saliency map. As described in the last section, we can locate the apron area firstly, and then we extract the candidate regions where the saliency indexes are calculated. The context information of apron and the candidate regions are important for accuracy and computational complexity of detection. In general, the entire aircraft detection procedure in high-resolution SAR images is illustrated in Figure 5.

Details of the steps are shown in the following:

(1) Enhance SAR image and remove image noise;
(2) Extract apron and non-apron area from the preprocessed image. In this step, we can obtain an apron area map and a map of candidate regions on the apron area;
(3) Obtain the original gradient image of the image by computing the horizontal and vertical gradient amplitude;
(4) Apply directional local gradient distribution over apron and candidate regions to compute the gradient textural saliency map;
(5) Segment the gradient textural saliency map with TL-CFAR algorithm. Finally, we detect the aircrafts located on the apron.

## 4. Experimental Results

The hardware environment of the experiments is the AMD Athlon(tm) Π X4 631 Quad-Core Processor 2.60 GHz, 4 GB of RAM; the software development environment is Microsoft Visual C++ 6.0 in Windows 7 of 32-bit operating system. In the experiments, we use real-world X-band airborne SAR images provided by Beijing Institute of Mechanical and Electronics Engineering, and the resolution for both range and azimuth is 0.5 m. All of them are the magnitude-detected images converted from the single-look complex-valued SAR images. In addition, the detectors for comparison are: VI-CFAR [13], SCCA-CFAR [13], TL-CFAR detectors [15], LED detector [23] (here we use it over apron and candidate regions), and Gradient Textual Saliency Map over apron and candidate regions (GTS detector).

For quantitatively evaluating the performance of a detector, the receiver operator characteristic curve (ROC curve) is usually adopted. Given a threshold for a detector, the pixel number of true positive (TP) is calculated by comparing the detection target with the ground truth mask, and the pixel number of false positive (FP) is calculated similarly. Suppose the pixel number of the ground truth mask is GTP, then we can compute *true positive rate* (TPR) and *false positive rate* (FPR) as:

(13) $$\text{TPR}=\;\frac{\text{TP}}{\text{GTP}},\text{ FPR}=\;\frac{\text{FP}}{\text{GTP}}$$

Furthermore, the ROC curve is plotted with the pairs of TPR *vs.* FPR.

**Figure 6:**
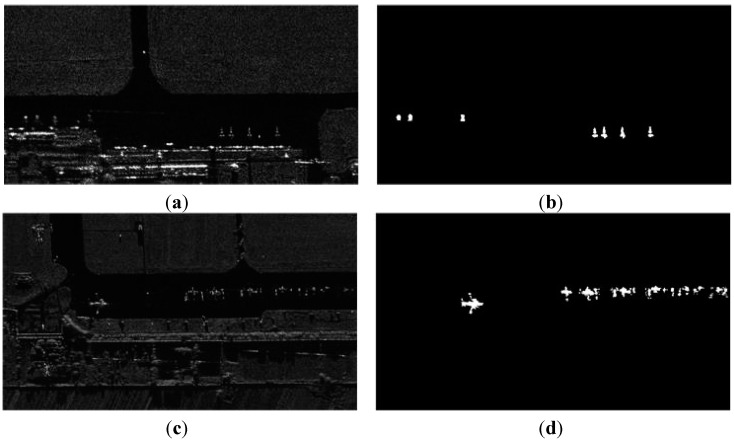
The ground truth examples of airplane targets in SAR images. (**a**,**c**) are the original SAR images; (**b**,**d**) are the ground truth targets labeled by hand.

For the final evaluation of detectors, we draw the ground truth of airplane targets in the original SAR images. Figure 6 shows the examples of the ground truth mask of airplane targets. In order to give the overview of the apron, we show the images at down-sampled resolution.

### 4.1. Determination of 4-Direction of Local Window

**Figure 7:**
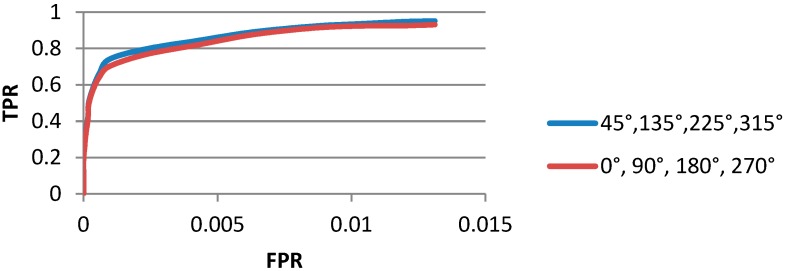
ROC curves of the two groups of directions. The red one is the ROC by using window of 0°–270° with 90° interval; the blue one is the ROC curve by using window of 45°–315° with 90° interval.

**Figure 8:**
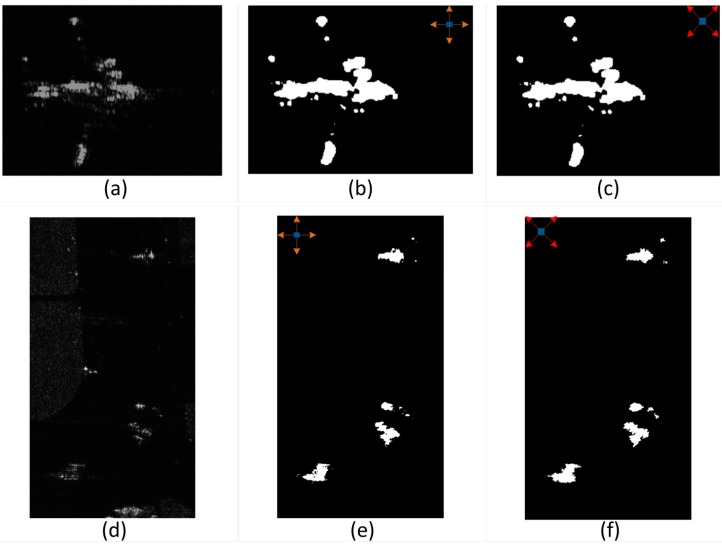
Different local 4-direction window and detection result. (**a**,**d**) are the original SAR images; (**b**,**e**) are the resultant images by using window of 0°–270° with 90° interval; (**c**,**f**) are the resultant images by using window of 45°–315° with 90° interval.

Different local 4-direction windows produce different detection results. For discrete image, here we compare two kinds of local window, one is with four direction of 45°, 135°, 225°, 315° and the other is 0°, 90°, 180°, 270°. In addition, window’s radius is 8 pixels for this experiment. In order to determine the best one between the two directions, we draw the ROC curve to reveal the performance. From the quantitative comparison demonstrated in Figure 7, it is obvious that the direction 45°, 135°, 225°, 315° is better. Meanwhile, we can also decide the direction from the view of visual perception. The final detection results are shown as Figure 8.

Figure 8 shows the detection results which are detected by different local 4-direction window. Figure 8a,d are the original images. Figure 8b,e show the results of window of 0°, 90°, 180°, 270°, and Figure 8c,f shows the results of window of 45°, 135°, 225°, 315°.

From Figure 8 we can see that Figure 8c,f have more complete and interconnected targets than Figure 8b,e. Figure 8b,e seem losing some details especially in the marginal area. However, Figure 8c,f almost have the same result in integrity and target structural area. Therefore, here we choose window of 45°, 135°, 225°, 315° rather than window of 0°, 90°, 180°, 270° in our situation.

### 4.2. Determination of Radius of Local 4-Direction Window

**Figure 9:**
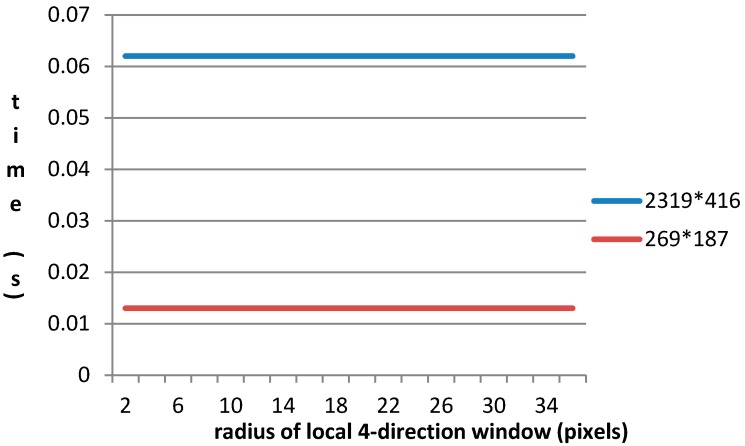
Relationship between the radius of local window and time of the detection algorithm.

From Equation (2), we can see that the value of the gradient textural saliency index is determined by $G_{de}$ and $G_{ev}$ as for the calculation process. It tells that $G_{s}$ is sensitive to the window’s radius. In this experiment, we use the images with size of 2319 × 416 and 269 × 187 pixels respectively. Figure 9 shows the relationship between the radius of local window and time of detection algorithm under the situation of steady objective conditions.

From Figure 9, we can conclude that, running time is insensitive to the window’s radius even though detection’s computation complexity increases while the radius increases. In addition, time consumption is basically around a small value, in case of size of 2319 × 416, it is 0.062 s, and in case of size of 269 × 187, it is 0.013 s. In addition, it is clear that, running time is sensitive to size of image, because detection’s computation complexity increases while the size of image increases.

**Figure 10:**
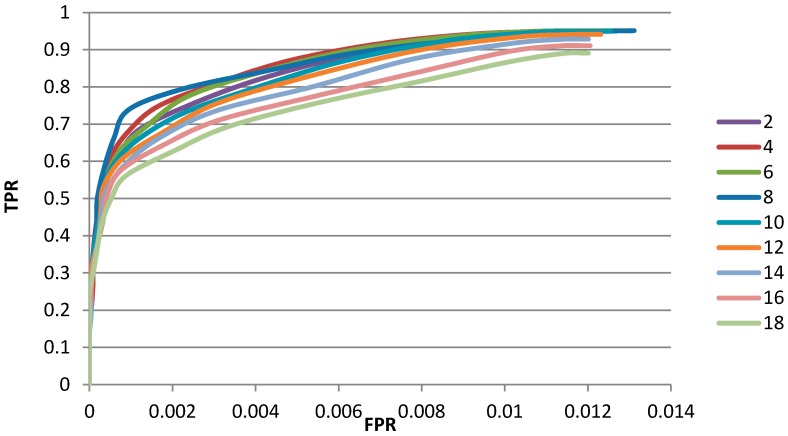
ROC curves by using different radius of the window (the unit of radius is pixel).

On the other hand, the different radius of local 4-direction window has different impact on the final detection result. The determination of radius can be considered from two aspects: quantitatively and qualitatively. For the quantitative determination, we draw the ROC curve for different radius, which is illustrated as Figure 10. The top curve is corresponding to the radius of 8 pixels.

**Figure 11:**
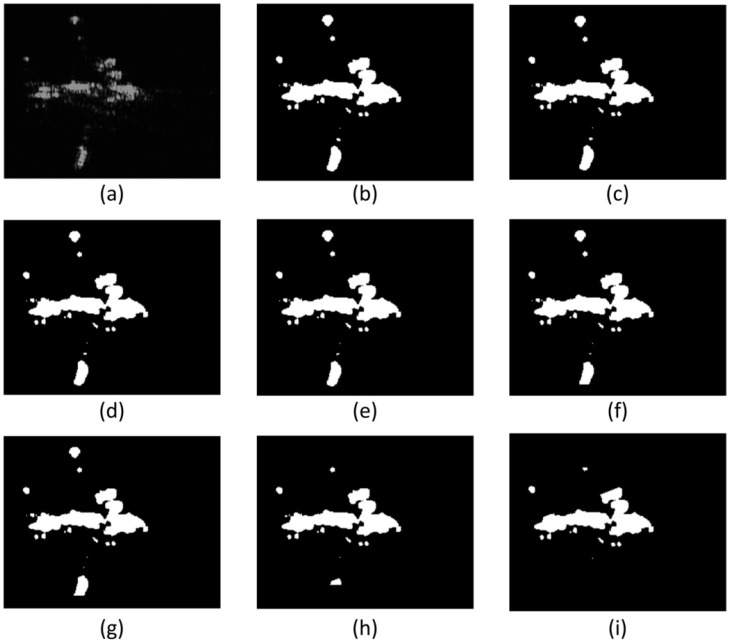
Radius of local window’s influence on detection result. (**a**) is the original image for example; (**b**) is the result of window with radius of 2 pixels; (**c**) is the result of window with radius of 4 pixels; (**d**) is the result of window with radius of 6 pixels; (**e**) is the result of window with radius of 8 pixels; (**f**) is the result of window with radius of 10 pixels; (**g**) is the result of window with radius of 12 pixels; (**h**) is the result of window with radius of 24 pixels; (**i**) is the result of window with radius of 36 pixels.

To give an intuitive explanation, the airplane detection results by using different window radius are shown as Figure 11. The visual relationship between radius of local 4-direction window and the detection result. In this experiment, we use TL-CFAR to segment the gradient textural saliency map. We can see that, the GTS-based detector can get better result when the radius is smaller than 12 pixels. In addition, shown as Figure 11 we can conclude that: the detection results are almost the same when the radius is smaller than 12 pixels, and the detection results miss details of the target when the radius is too big.

From the quantitative and qualitative comparison results, we set the radius 8 pixels in the following experiments.

### 4.3. Detection Performance

In this section, we compare the performance of the detectors quantitatively using ROC curves. In order to show the detected result intuitively, we compare the detected target for different detectors in three cases: multiple targets existing in apron, one target existing in apron and non target case.

#### 4.3.1. Quantitative Performance Comparison

**Figure 12:**
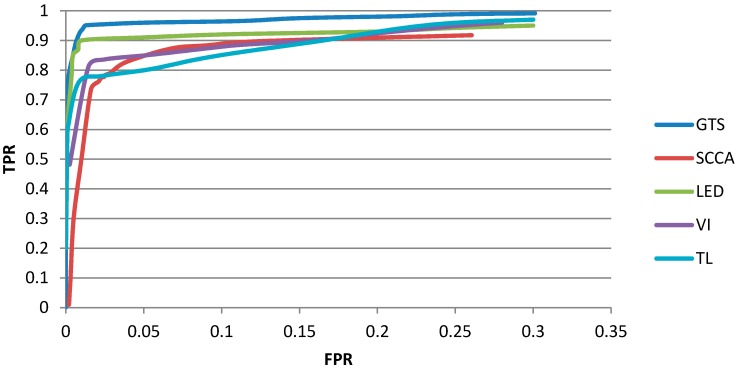
The ROC curves of the detectors for comparison.

In order to compare the performance of different detector objectively, we draw the ROC curves for different detectors by varying their detecting parameters. The ROC curves are shown as Figure 12. Generally speaking, all the detectors have such property that TPR converges very quickly as FPR increases.

Among the five detectors, GTS and LED are superior than the other algorithm. Figure 12 shows the GTS and LED have the similar curve shape even though the curve of GTS is better than that of LED. The reason of the similar shape is possibly that both GTS and LED describe the textural structure to characterize the target and are conducted on the same candidate regions. However, the GTS algorithm can describe the structure information better than the LED because the structure description of GTS is operated on the gradient domain while the LED rely on the edge detection result. This may explain the performance of GTS is better than LED.

Overall, the GTS achieves the best performance among the five detectors, which demonstrates the performance advantage of our proposed GTS detector.

In the following parts, we will visually analyze the performance of structure retention and object integrity for different detectors including the GTS-based detector in three cases: multi-targets, one-target and non-target situation.

#### 4.3.2. Visual Performance in Multi-Target Case

**Figure 13:**
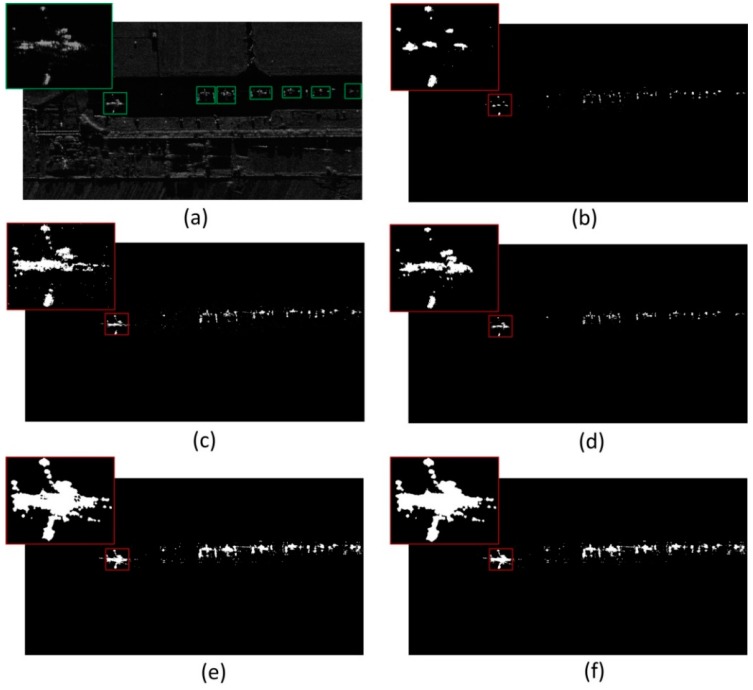
Results of different detectors in multi-targets case. (**a**) is the original SAR image; (**b**) is the result of VI-CFAR detector; (**c**) is the result of SCCA-CFAR detector; (**d**) is the result of TL-CFAR detector; (**e**) is the result of LED detector; (**f**) is the result of GTS detector. The regions labeled by green rectangles in (**a**) are the aircraft targets in the SAR image (**a**). In addition, the images on the top left corner are the original resolution of the regions which are labeled by red rectangle in the corresponding images. Specially, such region is labeled by a green rectangle in (**a**).

**Figure 14:**
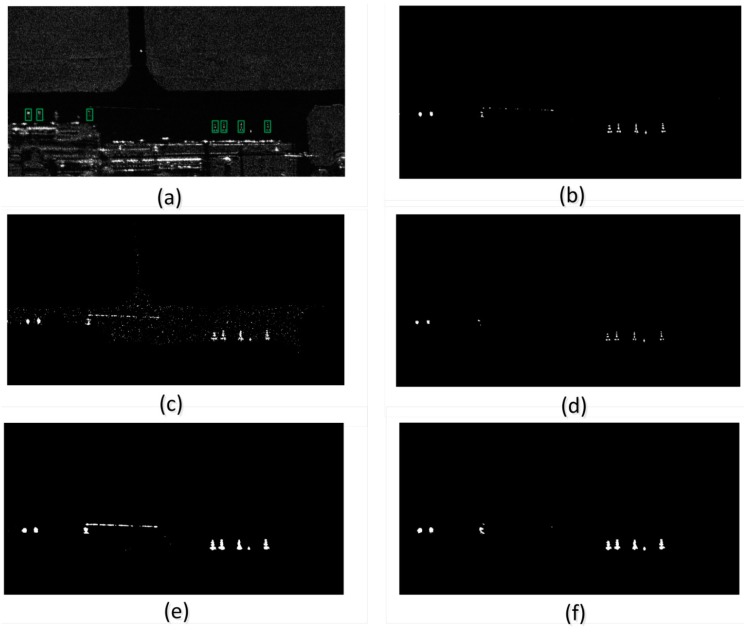
Results of different detector in multi-targets case. (**a**) is the original SAR image; (**b**) is the result of VI-CFAR detector; (**c**) is the result of SCCA-CFAR detector; (**d**) is the result of TL-CFAR detector; (**e**) is the result of LED detector; (**f**) is the result of GTS detector. The regions labeled by green rectangles in (**a**) are the aircraft targets in the SAR image (**a**).

Before we detect the targets, we apply the Frost filter to suppress the speckle noise on the original image, the width of the sliding window, the attenuation factor and the standard deviation of noise of the Frost filter are respectively 5 pixels, 5, and 0.5 in our experiment as empirical value. The original SAR images which contain multiple aircraft targets is shown as Figure 13a and Figure 14a. In the experiment, we set ${\text{P}}_{\text{fa}}=\;10^{-4}$ for the CFAR detectors except for TL-CFAR algorithm following the configurations of the references, where ${\text{P}}_{\text{fa}}$ is the constant probability of false alarm for CFAR detector. In addition, we set ${\text{P}}_{\text{fa}1}=\;10^{-3}$ and ${\text{P}}_{\text{fa}2}=\;10^{-4}$ for TL-CFAR detector according to [15]. Besides, the statistical models of CFAR algorithms follow normal distribution. The outer width of referential window is 21 pixels, and the width of the protection window is 20 pixels. In addition, we set the circle window’s radius 8 pixels for the proposed algorithm of GTS-based detector. The result will be different while this radius changes. In addition, this radius can be changed when the type of the target or the scene changes. Here these parameters’ values are set to satisfy both real-time performance and effectiveness. In addition, what’s more, these detectors only detect the targets on the apron area.

The results of different detectors under the same apron are shown in Figure 13 and Figure 14.

Except for the practical visual results of all the detectors, we also can look over the results in terms of quantitative analysis. Table 1 gives us the specific values of TPR and FPR when we set the parameters specified in the section.

As can be seen, GTS has the biggest TPR, and SCCA-CSAR has the biggest FPR. The numerical values can be combined with the visual results appearing in Figure 13 and Figure 14 for the performance analysis of the detectors.

We can conclude from Figure 13 and Figure 14 that VI-CFAR cannot get a good result in the multiple-target situation. In addition, from Figure 13b and Figure 14b we can see that the detected targets images are too scattered to be distinguished as intact aircrafts, because they miss many detailed pixels. In addition, VI-CFAR detector needs much prior information. SCCA-CFAR, TL-CFAR can get better results here. The targets are more intact than the previous mentioned detector. However, from Figure 13c,d and Figure 14c,d, we can see that: SCCA-CFAR detector’s global false alarm rate is the highest because its results contain many false alarm pixels which distract on the apron area as points or small regions; TL-CFAR detector gets a better result than other methods, but its results miss too many details, especially important intact structure and texture feature of the target, even though its results contain the least false alarms. While the proposed detector gets the best result here. From Figure 13e,f and Figure 14e,f, we can see that: LED detector and GTS detector can get better results than the other detectors here. They are good at keeping the resultant targets intact and remain legible and intact structure and texture feature. However, LED detector contains many false alarms, or cannot keep a target uniform fully connected. GTS detector can get intact targets, while it basically without false alarms. Clear details of resultant target and little false alarms are very important for a detector’s performance. In addition, the main bodies of the targets are clear with some small false alarm regions. In addition, the results of GTS contain the comparative small false alarms in our all experiments among all the detectors. According the practical imaging condition, we can remove the false alarm by the constraint of areas if there are some small target regions.

**Table 1:** Detection Results on Test Images.

Detector	VI-CFAR	SCCA-CFAR	TL-CFAR	LED	GTS
TPR	0.51	0.62	0.68	0.79	0.91
FPR	0.001	0.016	0.001	0.002	0.001

**Figure 15:**
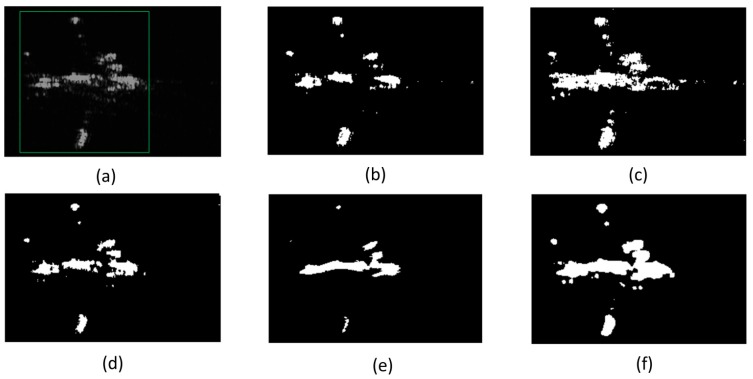
Results of different detector in condition of one-target situation. (**a**) is the original image for example; (**b**) is the result of VI-CFAR detector; (**c**) is the result of SCCA-CFAR detector; (**d**) is the result of TL-CFAR detector; (**e**) is the result of LED detector; (**f**) is the result of GTS detector. The region labeled by a green rectangle in (**a**) is the aircraft target in the real SAR image patch (**a**).

To conclude, GTS-based detector can keep the parts of an aircraft combine tight enough, keep a target uniform fully connected and meanwhile keep the false alarm rate in a tolerable allowance. Figure 13e,f may not show the results so clearly different between LED-based detector and GTS-based detector, but Figure 14e,f and the next part’s Figure 15e,f show the results’ difference clearly.

#### 4.3.3. Visual Performance in One-Target Case

The parameters here are set same as the multi-target case. The results of different detectors are shown in Figure 15. Shown as Figure 15, all the other detectors here for comparison cannot get a better result than GTS-based detector proposed in this paper under the one-target situation. TL-CFAR gets a very scattered target parts. LED-based detector gets targets with missing many details. The GTS-based detector can get a very intact target at a low false alarm rate, as Figure 15f shows.

#### 4.3.4. Visual Performance in Non-Target Condition

**Figure 16:**
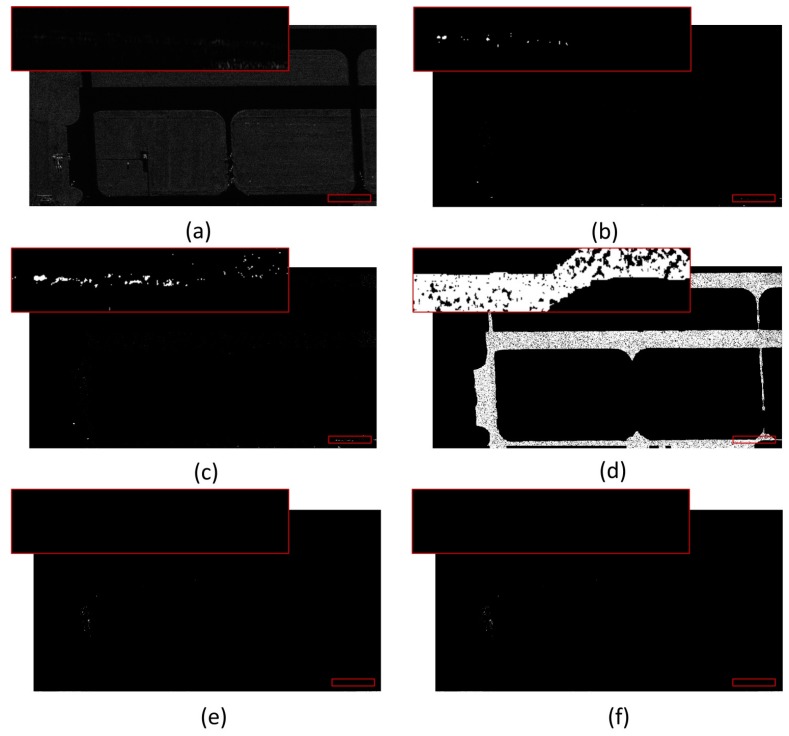
Results of different detectors in case of non-target. (**a**) is the original image for example; (**b**) is the result of VI-CFAR detector; (**c**) is the result of SCCA-CFAR detector; (**d**) is the result of TL-CFAR detector; (**e**) is the result of LED detector; (**f**) is the result of GTS detector. In addition, the images on the left top corner of all the images are the detailed drawings of the regions which are labeled by red rectangles in the corresponding images.

In this part, we will analyze the performance of different detectors under non-target situation. The parameters here are set same as multi-target case. The results of different detectors are shown in Figure 16. Shown as Figure 16, SCCA-CFAR, TL-CFAR cannot get a better result than GTS-based detector proposed in this paper, except for VI-CFAR because the background of the image is uniformly distributed. From the Figure 16d we can see clearly that TL-CFAR gets a very high false alarm rate than the other detectors with very scattered target parts. SCCA-CFAR gets a better result than TL-CFAR, but it still obtains very scattered target points all over the apron area, even though we cannot see the points all over the apron area because the resultant image is too small and low-resolution. In addition, VI-CFAR can get better result than SCCA-CFAR, but it still has a relatively higher false alarm rate than Figure 16e,f with relatively scattered points over the apron area which we cannot see clearly here. From Figure 16e,f ,we can see that, LED-based and GTS-based can get better results. However, the GTS-based detector can still get a relatively good result here with a relatively lower false alarm rate. Therefore, we can conclude that GTS-based detector can obtain good results in most cases.

### 4.4. Computing Time

Figure 16 is the running time of several kinds of detectors under the same condition. The image with size of 269 × 187 contains one aircraft target. The image with size of 735 × 552 contains thirteen aircraft targets. The image with size of 559 × 188 contains five aircraft targets on its apron area. The image with size of 2800 × 1558 contains six aircraft targets on its apron area.

**Figure 17:**
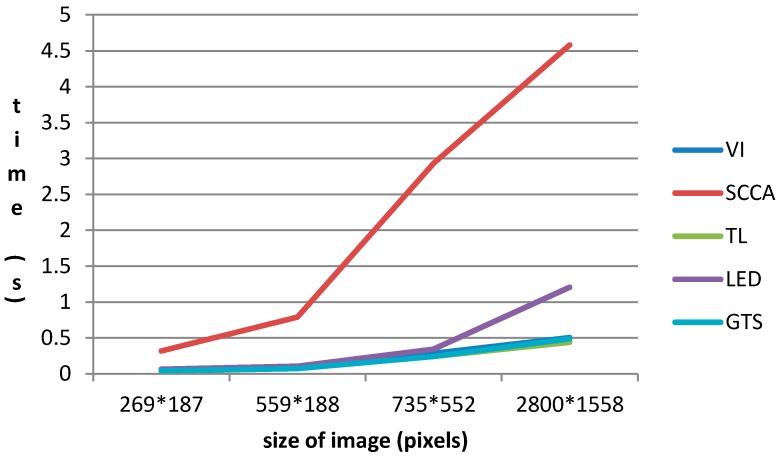
Running time of different detectors.

From Figure 17 we can see that: the detectors’ time consumptions are listed in descending order as following according to detectors’ computation amount: SCCA-CFAR, LED-based detector, VI-CFAR, GTS-based TL-CFAR detector, TL-CFAR detector. In addition, the running time of these detectors increases as the image size increases. In addition, with the scene’s complexity and the number of targets increase, the time consumption increases rapidly. The algorithm computation complexity of GTS-based will increase while the scene’s complexity and the gradient image’s complexity increase. However, GTS-based method’s very simple calculation makes it get correct results with very few calculations. Meanwhile, the strategy of applying apron image and candidate region map can do a great favor to reduce the computation complexity in step of the saliency map’s generation. Therefore, GTS-based detector can meet the real-time requirement.

## 5. Conclusions

In this paper, we proposed an aircraft detection algorithm in high-resolution SAR images based on gradient textural saliency map and contextual cues. The contextual cues that aircraft locates in apron and appears as a hole when detecting apron, can largely reduce the computational complexity because we can analyze gradient textural saliency map only in the local area indicated by the hole. In addition, the algorithm obtains much more precise and intact information of the aircraft targets compared with the other algorithms. The experimental results based on the real SAR images have validated that the proposed GTS-based algorithm is effective and practicable.

As the front-end input for the automatic target recognition (ATR) system, the improvement of detection accuracy to overcome the discontinuity of target in SAR image needs to be further studied. The strategies include developing new feature descriptor of target, constructing new model of contextual knowledge, and exploiting the distribution distinction between target and background clutter. For practical application, the optimization of detection algorithms to satisfy the computational constraints on running time, memory capacity and configuration of embedding system is also a big challenge.
